# Impaired Iron Status in Aging Research

**DOI:** 10.3390/ijms13022368

**Published:** 2012-02-22

**Authors:** Jinze Xu, Zhenhua Jia, Mitchell D. Knutson, Christiaan Leeuwenburgh

**Affiliations:** 1 Department of Aging and Geriatric Research, College of Medicine, Institute on Aging, University of Florida, Gainesville, FL 32611, USA; 2 Biology Institute, Hebei Academy of Science, Shijiazhuang 050051, China; E-Mail: zhenhuaj@hotmail.com; 3 Food Science and Human Nutrition Department, University of Florida, Gainesville, FL 32611, USA; E-Mail: mknutson@ufl.edu

**Keywords:** labile iron, iron accumulation, oxidative damage, mitochondrial dysfunction, aging

## Abstract

Aging is associated with disturbances in iron metabolism and storage. During the last decade, remarkable progress has been made toward understanding their cellular and molecular mechanisms in aging and age-associated diseases using both cultured cells and animal models. The field has moved beyond descriptive studies to potential intervention studies focusing on iron chelation and removal. However, some findings remain controversial and inconsistent. This review summarizes important features of iron dyshomeostasis in aging research with a particular emphasis on current knowledge of the mechanisms underlying age-associated disorders in rodent models.

## 1. Introduction

Iron is an essential nutrient. Disturbances of iron metabolism may have deleterious consequences in severe pathological conditions such as cardiovascular diseases [1–3], diabetes [4], cancer [5–7] and neurodegenerative diseases [8–12]. It has been widely documented that aging is associated with dyshomeostasis of iron metabolism and regulation in both rodents [13–25] (Table 1) and humans [26–32]. The elderly are more prone to becoming anemic [33–37], which adversely affects muscle strength [38], physical performance [39], cognition [40] and longevity [41]. In contrast, age-related iron overload is also increasingly being recognized as a public health concern [42–45]. Despite the prevalence and adverse health effects associated with these disorders, the mechanisms are still not well defined and many questions remain to be answered [2,46,47].

## 2. Impaired Iron Status with Age in Rodent Models

### 2.1. Organ-Specific Changes in Iron Content with Age

During the last decade, a number of studies have documented age-related iron accumulation in rodents (Table 1). One of the earliest studies (by Massie *et al.* [20]) revealed age-related changes in iron content in young (1.5–7 months), middle-aged (21 months) and aged (30 months) male C57BL/6J mice. They showed that total iron concentrations were significantly elevated in the liver, heart, kidney and brain of aged animals. In further support of the finding of iron dyshomeostasis in aging, Sohal *et al.* [24] reported that there was an age-associated increase in non-heme iron levels in liver, kidney, brain and heart, which, however, is independent of the increases in redox-active iron determined using bleomycin-detectable iron assay. In addition, the observation in the same study that life-long 40% caloric restriction had no effect on iron levels in heart and brain and even exacerbated iron accumulation in liver and kidney does not support the hypothesis that labile iron plays an essential role underlying the age-associated increase in oxidative damage. On the contrary, a successful attempt to ameliorate age-related iron accumulation by life-long 40% caloric restriction was published by Cook and Yu [19] in 1998. The results of their study in male Fischer 344 rats showed a remarkable age-related increase in non-heme iron levels in liver, kidney and brain of animals fed *ad libitum*. Their finding that caloric restriction markedly mitigated iron accumulation in multiple tissue systems of aged animals as well as our recent study [17] suggests that caloric restriction beneficially modulates iron dyshomeostasis.

Since iron accumulation is widely accepted as a feature of the aging process particularly in post-mitotic tissues by emerging research in the intervening decade [14–18,21,22,25], a substantial research effort has been directed at exploring potential iron chelation therapies. Recently, deferiprone and deferasirox emerged as promising orally active iron-sequestering agents [23,48,49]. Arvapalli *et al.* [21] reported that deferasirox, administrated at a dose of 100 mg/kg body weight on alternate days for 6 months, was effective in reducing total iron levels in the heart and liver as well as attenuating cardiomyocyte apoptosis in 27-month-old Fischer 344 x Brown Norway rats. However, limited information is provided in the same study to warrant that the use of chelator did not exacerbate the low serum ferritin usually observed in aged animals. A non-toxic iron chelator or potential treatment strategy that locally removes excess iron in particular tissues without affecting systemic iron utilization, storage and transport, may represent an ideal therapeutic intervention.

In contrast to the above-mentioned studies, Ahluwalia *et al.* [13] showed that total non-heme iron levels in the liver, spleen, and bone marrow of Lewis rats declined with age. The conflicting study findings may stem from several factors involved in rodent aging research, such as strain, species, diets, and life stages of animals.

### 2.2. Life Stage, Species, Sex and Strain Differences across Studies

Though research scientists have made considerable progress in defining rodent life stages across species and strains, crucial definitional problems remain unsolved. It could be considered as a central challenge in investigating age-associated changes in iron homeostasis and metabolism primarily because the average lifespan varies greatly depending on sex, strain, and breeding system.

Outbred strains, such as Sprague Dawley and Wistar rats, have been widely used to investigate iron homeostasis and metabolism in aging research, while C57BL/6 mice, Fischer 344 rats, and Lewis rats as inbred strains are excellent models as well. Recently, Fischer 344 x Brown Norway rat, a F1 hybrid strain, has been proposed as a potential model for aging since it most closely reproduces healthy aging in humans [55,56]. A comparative study on the muscle mass and contractile properties between Fischer 344 x Brown Norway and Fischer 344 rats by Rice *et al.* [55] indicated that there were ageassociated decreases in both of the two sub-populations of muscle fibers in Fischer 344 x Brown Norway rats, suggesting that the F1 hybrid strain is a better model of sarcopenia than Fischer 344. Besides the differences in age-associated physiological or pathological alterations, outbred and F1 hybrid animals exhibit hybrid vigor with long lifespans. The median survival ages for male and female Fischer 344 rats are 24 and 26 months, respectively; however it extends to 34 months for male and 30 months for female Fischer 344 x Brown Norway rats [50]. In the basic science of aging, two or three age cohorts were commonly selected and referred to representative life stages as young and old animals or young, middle-aged, and old animals, which may dramatically limit the power of investigations and the universality of conclusions. If indeed iron is a contributing factor in the aging process, a significant alterations in iron levels or metabolism between young and aged animals will be detectable at the point that iron dyshomeostasis has occurred in the study population and remains relatively stable. Given the fact that aged rats and mice past the 25% survival age are more prone to underlying age-associated diseases and are not a good research model of healthy aging for most purposes, the cut-off age at which point iron status has substantially altered while the incidence of pathologies is relatively low is crucial in aging research using rodent models.

Table 1 lists the animal studies reporting organ-specific changes in iron content with age in rodents. The median survival age of each strain has been employed to estimate and compare animal life stages across studies. Aged cohorts at their median survival ages have been included in these studies except for the one provided by Takeda *et al.* [51], who reported an age-related iron accumulation using young (3-week-old) and mature (6-month-old) Wistar female rats. However, other investigators using male Fischer 344 [19] and male Fischer 344 x Brown Norway rats [17] indicated that iron levels in liver remained unchanged until late middle age. Recently, Hahn *et al.* [13,57] reported age- and sex-dependent changes in tissue iron levels among C57BL/6, DBA/2J, and BALB/c mouse strains and further confirmed that there were age-dependent and sex-specific changes in mouse tissue iron by strain. Taken together, these observations suggest the onset of impaired iron status in rodent models highly depends on sex and strain.

### 2.3. Age-Associated Decrease in Heme Iron Levels *vs.* Increase in Non-Heme Iron Levels

Measurements of iron levels in rodent models usually fall into two categories: total iron determined by spectrometry techniques, such as atomic absorption spectroscopy [20] or inductively coupled plasma emission spectrometry [21,51], and non-heme iron measured by colorimetric methods [13–19]. Total iron includes both heme and non-heme iron. The first indication that heme biosynthesis declines with age was provided by Bitar and Weiner [58], who examined age-related changes in heme and heme proteins in male Sprague-Dawley rats. This finding was further confirmed by studies with emphasis on heme deficiency in both neurodegenerative disorders [59–62] and normal aging [19]. In considering the findings of age-associated decline in heme biosynthesis and increases in non-heme iron levels, total iron measurements *per se* may not fully reflect iron dyshomeostasis in aging research, in particular when the conclusion of unaltered iron levels over time was reached using spectrometry techniques.

### 2.4. Ferroportin—The Only Way out for Cellular Iron

**Figure 1:**
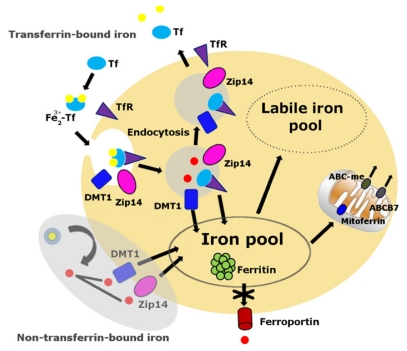
Cellular labile iron pool. The transferrin-transferrin receptor (Tf-TfR) pathway is the primary route of cellular iron acquisition [67]. Cells assimilate iron when Fe^3+^-Tf binds to TfR at the cell surface, and the complex is internalized into endosomes. Endosomal acidification promotes iron to dissociate from Tf, and the metal is then reduced to Fe^2+^ and transported into the cytosol by the transmembrane protein divalent metal transporter 1 (DMT1) and Zip14. The non-Tf-bound iron pathway, the shaded area, appears mainly during states of iron overload. Much of the iron normally assimilated by cells is destined to the mitochondria via mitoferrin, the site of heme and iron-sulfur cluster biosynthesis. Iron is exported from the mitochondria in the form of iron-sulfur clusters or heme. Export of iron-sulfur clusters involves ABCB7. Cells export iron through ferroportin. The absence of ferroportin in skeletal muscles and other post-mitotic tissues may result in iron accumulation over time.

Cellular iron balance is coordinated by iron uptake, storage and export [63–65]. Iron cannot diffuse through cellular membranes unassisted. Either a receptor-mediated or non-receptor-mediated pathway is required to facilitate cellular iron import into the cytoplasm (Figure 1). The primary route of cellular iron acquisition is through receptor-mediated endocytosis of transferrin (Tf) [66]. Cells take up Tf-bound iron in proportion to their cell-surface expression of transferrin receptor (TfR) [67]. Divalent metal transporter-1 (DMT1), a ferrous iron transporter, can import iron into the cell, a mechanism which is essential for intestinal uptake of inorganic sources of dietary iron [65]. Zip14, a member of the SLC39 metal-ion transporter family, has also been shown to mediate iron uptake by cells [68,69]. Cellular iron export is mediated by ferroportin, the only known iron exporter in mammals [70].

Ferroportin is a transmembrane protein expressed on the surface of absorptive enterocytes, macrophages, hepatocytes, and placental cells [70]. Tissue-specific ablation of ferroportin results in embryonic lethality [70]. McKie *et al.* [71] reported that ferroportin was weakly expressed in kidney, liver, and testis and absent in brain, heart, lung and skeletal muscle of mice fed on a normal diet [71]. A recent study on muscle iron metabolism in Fischer 344 x Brown Norway rats by us [15] further supported the finding that the absence of ferroportin in skeletal muscles significantly contributes to the iron accumulation in aged animals. Thus, the lack of a cellular iron export mechanism in post-mitotic tissues could be one of the essential factors contributing to iron accumulation in aging.

### 2.5. Animal Diets

Although iron balance is tightly regulated at the site of absorption (duodenum) [72], rodent diets with different iron levels may alter iron homeostasis across studies. The AIN-76 diet (American Institute of Nutrition, 1977) or AIN-93 [73], a substitute for the original AIN-76 diet to improve the performance of animals, is a widely used purified diet for laboratory rodents formulated with 35 mg iron/kg diet, an amount considered to meet the minimum requirement of iron for normal growth and hematopoiesis [74]. Natural-ingredient and typical rodent diets, which usually contain 198 to 270 mg iron/kg diet, have also been used to provide good health and reproduction in laboratory rodents [74,75]. In early 1970s, Sorbie and Valberg [76] observed that 25 to 100 mg iron/kg diet was associated with low iron storage in liver of male C57BL/6J mice and that higher concentrations may be necessary for reproduction. A recent study published by Cooksey *et al.* [77] also indicated that the mice on the 35 mg·iron/kg diet did exhibit remarkable decreases in hepatic iron and serum ferritin compared with mice on the 500 mg·iron/kg diet. In agreement with these observations, a long-term study on AIN-93M (maintenance formulation) diet by Ahluwalia *et al.* [13] showed that iron status and stores in liver, spleen and femur marrow decline with age in male Lewis rats. Despite the low iron stores in animals fed AIN purified diets, Jung *et al.* [22] demonstrated that non-heme iron and ferritin levels significantly increased with age in the plantaris muscle of male Fischer 344 rats during short-term feeding with AIN-93M diet, suggesting that skeletal muscles are extremely vulnerable to iron accumulation in aging. Long-term studies on dietary modification or adjustments are warranted to create an optimal diet containing a maintenance-level of iron that is suitable for rodent models at different life stages.

## 3. Iron Accumulation and Labile Iron

Cells maintain a pool of available labile iron [78] (Figure 1) identified by several terms, including “transition iron”, “free iron”, “low-molecular-weight iron”, “redox-active iron” or “chelatable iron” [79], which exists in dynamic equilibrium with various cellular components. Optimal function of cells highly depends on the maintenance of cellular iron levels [80]. When iron prevails over cellular iron sequestration, labile iron may be released from either loosely bound iron proteins or storage sites, particularly under conditions of cellular stress [81]. Labile iron is highly reactive and has the potential to catalyze the formation of harmful reactive oxygen species, ultimately leading to oxidative damage and cell death [82,83]. In light of previous studies showing catastrophic cellular damage by labile iron, Simunek *et al.* [84] showed that H_2_O_2_-induced collapse of mitochondrial membrane potential was completely prevented by pre-treatment with the lipophilic iron chelator, salicylaldehyde isonicotinoyl hydrazone (SIH), in cultured H9c2 cardiac myoblasts, suggesting that hydrogen peroxide *per se* is not harmful, but it may become highly toxic if labile iron coexists. Furthermore, the observation that iron chelation by an iron chelator, deferoxamine, mitigated immobilization-induced muscle loss in male Wistar rats implies that labile iron could be one of several potential contributors to accelerate muscle atrophy during prolonged inactivity [85].

## 4. Iron Dyshomeostasis in Age-Associated Disorders in Humans

Clinical and epidemiological studies have shown that iron plays important roles in multiple agingassociated disorders, such as cardiovascular diseases [1,2], inflammatory diseases [86], neurodegenerative diseases [10,11], and cancer [5–7,87]. Salonen *et al.* [88] reported that men with serum ferritin greater than or equal to 200 μg/L had a 2.2-fold increased risk of acute myocardial infarction compared with men with a lower serum ferritin at age of 42 to 60 in eastern Finland. In agreement with the epidemiological finding, Tuomainen *et al.* [89] demonstrated that men with high body iron stores were at a 2- to 3-fold increased risk of the first acute myocardial infarction. In healthy subjects [90] and anemic patients [91], the level of serum ferritin showed an age-related tendency to increase. Recently, Tull *et al.* [92] indicated that these subjects are likely to have anemia of chronic diseases with adequate iron stores and unable to utilize iron from storage sites. Therefore, the most common cause of anemia in the elderly is anemia of chronic disease, which has been identified as impaired iron status rather than iron deficiency.

Age-associated decline in hematologic variables has been the subject of extensive investigation in animal models [13,17,93–95] and humans [28,32,36,96]. A number of studies have shown that aging is associated with an erythropoietic decline [93,97] as well as a reduced reserve capacity in the hematopoietic system [98–100]. However, an early study reported by Boggs and Patrene [94] using B6D2 F1 female mice argued that an expanded plasma volume in aged animals substantially contributed to the decrease in hematocrit, whereas circulating red cell mass remained unchanged in aged animals, suggesting an age-related “dilutional” anemia. In a follow-up study using a mathematical model of erythropoiesis, Loeffler and Pantel [101] revealed that the lower hematocrits in aged mice were due to plasma volume expansion, rather than changes in red cell mass between young and aged animals. A clinical study of dietary iron intake and excretion in healthy elderly subjects aged 70 to 85 years indicated that hemoglobin levels were within the established reference range for adult individuals.

It has been proposed that age-related anemia may be associated with increased hepcidin levels in response to elevated interleukin-6 levels [102]. Hepcidin is the principal regulatory hormone produced by hepatocytes in response to iron loading [103] or inflammation [104]. Under iron overload conditions, hepcidin downregulates ferroportin expression in enterocytes and macrophages, thereby reducing serum iron levels via decreasing intestinal iron absorption and macrophage iron recycling [105]. Despite the important role of hepcidin in systemic iron homeostasis, quantification of plasma hepcidin has proved to be technically difficult. The development of the first validated serum enzyme-linked immunosorbent assay (ELISA) by Ganz *et al.* [106] has allowed significant advances in studies of ageassociated alterations in plasma hepcidin levels. A recent study using the ELISA assay in anemic patients by Lee *et al.* [107] showed that anemia in the elderly was not associated with increased plasma hepcidin levels. The observation in the same study that both the mean and median hepcidin levels were lower in anemic elderly patients suggests that elevated plasma hepcidin levels may be secondary to age-associated pathology, acute or chronic infections and inflammation. The findings further support the conclusion reported by Tull *et al.* [92] that aging is associated with impaired iron status, a most common cause of anemia in the elderly.

## 5. Iron and Mitochondrial Function in Aging

The mitochondrion is the central site of heme and iron-sulfur cluster biosynthesis [108]. Recent studies in both yeast and mammalian systems have shown that mitochondrial iron increase with age, in particular under conditions of cellular stress, which may be a potential causative factor in age-related mitochondrial dysfunction [18,109–111]. Rauen *et al.* [79] have developed a selective mitochondrial iron fluorescent probe, rhodamine B 4-[(2,20-bipyridin-4-yl)aminocarbonyl]benzyl ester (RDA), which shows that labile iron was about 16.0 μM in rat hepatocyte mitochondria. A study on muscle mitochondrial function in aged rats from our group [18] showed that aging was associated with elevated mitochondrial non-heme iron levels in skeletal muscle, which is significantly correlated with mitochondrial susceptibility to permeability transition pore opening, an important factor in the pathogenesis of cell death. Moreover, Veatch *et al.* [109] established a link between defects in ironsulfur cluster biosynthesis and genomic instability in yeast aging research. It has been shown that yeast cell aging was associated with an impairment of mitochondrial DNA integrity, which in turn affects the transport efficiency of iron-sulfur proteins between cytoplasm and mitochondria. Furthermore, impaired mitochondrial iron-sulfur biosynthesis contributed to increased cellular iron acquisition, iron regulon activation and mitochondrial iron accumulation. These observations highlight the mechanism of altered iron homeostasis in mitochondria, which may cause multiple defects in mitochondrial heme and iron-sulfur cluster biosynthesis as well as iron accumulation.

## 6. Future Research

Age-associated iron dyshomeostasis is a process of progressive changes in multiple organ systems. Much research effort is directed at developing therapeutics or interventions to combat these changes. Some impressive successes have been achieved in non-mammalian models using iron chelators to mitigate iron overload and iron-related disorders, such as Alzheimer’s disease [112,113], Parkinson’s disease [114,115], Friedreich’s ataxia [116,117] and retinal disease [118,119]. A major concern arises from iron chelation therapy against the aging process is that compounds available to date cannot specifically target individual organs or systems. This may dramatically limit the use of iron chelators in elderly persons, in particular when considering the finding that altered iron status is characterized by adequate iron stores and low hematologic variables in both rodent [17] and human studies [92,120].

A major research challenge will be to develop novel, safe and feasible interventions that mitigate age-associated iron dyshomeostasis. Indeed, calorie restriction has been shown to be effective in modulating the age-associated iron accumulation in rat muscle, liver, brain and kidney [17,19,121]. Late-onset caloric restriction has proven to be less effective [122,123]. Dietary compounds that inhibit iron absorption (e.g., polyphenols in tea and coffee [124,125]) may offer alternative approaches to mitigate iron accumulation during the aging process. Future research is warranted to test dietary interventions.

## 7. Conclusions

In summary, impaired iron status and iron dyshomeostasis are associated with organ-specific changes in iron levels in multiple organ systems with age. Lack of ferroportin expression, at least in part, exacerbates iron accumulation over time in various tissues, such as skeletal muscle. Future research can be directed to late-onset therapeutics or interventions for modulating impaired iron status in aging.

## Figures and Tables

**Table 1:** Summary of studies reporting organ-specific changes in iron content with age in rodents.

Reference	Species (sex)	Young (months)	Middleaged (months)	Old (months)	Median survival age (months)	Total iron or non-heme iron	Other measures	Increase with age	Decrease with age	No change	Intervention
Massie *et al.*, 1983 [20]	C57BL/6J mice (M)	1.5–7	21	30	27 [50]	Total iron *		Liver, Kidney Brain Heart			
Takeda *et al.*, 1996 [51]	Wistar rats (F)	0.75, 6			29 [52]	Total iron **		Brain Lung Heart Liver Spleen Kidney Muscle			
Cook and Yu, 1998 [19]	Fischer 344 rats (M)	6	12	24	24 [50]	Non-heme iron #		Liver Kidney Brain			Caloric restriction
							Hemoglobin		Kidney	Liver, Brain	
Sohal *et al.*, 1999 [24]	C57BL/6 mice (M)	4, 8.5	17	27, 30	27 [50]	Non-heme iron #		Liver Kidney Brain Heart			Caloric restriction
Ahluwalia *et al.*, 2000 [13]	Lewis rats (M)	2–3	8–10	20–22	24 [53]	Non-heme iron #			Liver, Spleen, Femur marrow		
							Hemoglobin, Hematocrit, Plasma iron		Blood		
Altun *et al.*, 2007 [14]	Sprague- Dawley rats (M)	4		30	21 [54]	Non-heme iron #		Skeletal muscle			
							Transferrin	Skeletal muscle			
Jung *et al.*, 2007 [22]	Fischer 344 rats (M)	6		24–26	24 [50]	Non-heme iron #		Skeletal muscle			
							Ferritin	Skeletal muscle			
							TfR		Skeletal muscle		
Xu *et al.*, 2008 [17]	F344xBN rats (M)	8	18	29, 37	34 [50]	Non-heme iron #		Skeletal muscle Liver			Caloric restriction
							Hemoglobin Hematocrit		Blood		
Hofer *et al.*, 2008 [16]	F344xBN rats (M)	6		32	34 [50]	Non-heme iron #		Skeletal muscle			
							Free iron	Skeletal muscle			
							TfR	Skeletal muscle			
Seo *et al.*, 2008 [18]	F344xBN rats (M)	8	18	29, 37	34 [50]	Non-heme iron #		Muscle mitochondria			
Arvapalli *et al.*, 2010 [21]	F344xBN rats (M)	6		27	34 [50]	Total iron **		Heart Liver			Deferasirox 100 mg/kg BW for 6 months
Bulvik *et al.*, 2011 [25]	Wistar rats (F)	2		24	29 [52]	Ferritinbound iron		Spleen, Liver, Tongue, Sternohyoid		Esophagus	
Xu *et al.* 2011 [15]	F344xBN rats (M)	6		32	34 [50]	Non-heme iron #		Skeletal muscle			
							TfR		Skeletal muscle		
							DMT1			Skeletal muscle	
							Zip14			Skeletal muscle	

Abbreviation: F344xBN rats, Fisher 344 x Brown Norway rats; DMT1, divalent metal transporter-1; TfR, transferrin receptor; (M), male, (F), female.

* measured by atomic absorption spectroscopy;

** measured by inductively coupled plasma emission spectrometry;

# measured by colorimetric method.
